# Alexithymia and Treatment Outcome in Anorexia Nervosa: A Scoping Review of the Literature

**DOI:** 10.3389/fpsyt.2019.00991

**Published:** 2020-02-14

**Authors:** Carla Gramaglia, Eleonora Gambaro, Patrizia Zeppegno

**Affiliations:** ^1^ Institute of Psychiatry, Università degli Studi del Piemonte Orientale, Novara, Italy; ^2^ S.C. Psichiatria, Azienda Ospedaliero Universitaria Maggiore della Carità, Novara, Italy

**Keywords:** alexithymia, anorexia nervosa, systematic review, PRISMA, treatment, outcome

## Abstract

Alexithymia is of great interest as an outcome predictor of recovery from anorexia nervosa, since it may interfere with both treatment compliance and patients’ ability to benefit from the adopted interventions. For this reason, in the last years new treatment approaches targeting emotion identification, expression, and regulation have been applied and tested. Using the PRISMA methodology, we performed a scoping review of the literature about treatment outcome in anorexia nervosa, in terms of changes in alexithymia as assessed by its most commonly used self-report measure, the Toronto Alexithymia Scale (TAS). The Medline and Scopus databases were searched, and articles were included if matching the following criteria: dealing with patients affected by anorexia nervosa, without limits of age; involving the application of any kind of targeted therapy or treatment; assessing alexithymia and the effect of a treatment intervention on alexithymia, using the TAS. Ten studies were eventually included; overall, according to the selected studies, alexithymia levels often remain high even after specific treatment. Further research aimed at a deeper understanding of the actual impact of alexithymia on the outcome of anorexia, as well as exploring alternative treatment strategies for alexithymia in eating disorders (EDs), are warranted.

## Introduction

Alexithymia is defined as a difficulty and inability in identifying and describing feelings and emotions (1): alexithymic individuals usually show a paucity of words to describe their affective status, and find it difficult distinguishing feelings from physical sensations. Furthermore, alexithymia is characterized by a diminution of fantasy and a concrete and externally-oriented thinking style (2). It is likely a stable personality trait reflecting an impairment of emotion regulation (3, 4), rather than a state-dependent phenomenon linked to depression or to clinical status (5, 6).

Impaired emotional functioning and alexithymic traits are core features of anorexia nervosa, both in the adolescent and adult population (7–15): they seem to be independent from depressive symptoms and eating disorder severity (16–18) and express themselves as a concrete, reality-based cognitive style and a poor inner emotional and fantasy life.

A “cognitive-affective” division (19) has been suggested to describe the experience of patients with eating disorders (EDs) when trying to translate their thoughts at a cognitive level into what is felt from an emotional standpoint. The emotional difficulties of individuals with anorexia nervosa (AN) may not depend on a primary impairment in emotion recognition, but rather on inhibition or avoidance occurring after a proper acknowledgment of others’ emotions (20), possibly due to patients’ feelings of being overwhelmed by over-control and anxious worry (21). Alexithymic traits in AN may cause patients avoiding or regulating the experience of emotions, especially those perceived as negative or disruptive (22), with inappropriate behaviors (restriction, binges, purges, body checking behaviors) (23), leading to a gap between the inner experience of negative affect and its expression (13). Actually, also in a non-clinical sample of undergraduate women, alexithymic individuals, compared to non-alexithymic ones, had more body checking behaviors, greater body dissatisfaction, and higher potential risk for EDs (24).

Implications at the social level of alexithymia and emotional difficulties (such as emotional avoidance and poor regulation), include low social emotional intelligence (25), problems with intimacy, attachment and social communication or interactions (26–29), and social anhedonia (30, 31).

Moreover, alexithymia and emotion regulation difficulties have been shown to have an impact on the course and maintenance of anorexia (12, 15, 32, 33), and on treatment outcome and recovery (34, 35). Actually, the lack of insight and the externally-oriented thinking style typical of alexithymia may interfere with treatment compliance and with patients’ ability to benefit from interventions, especially psychotherapy ones (36).

According to these clinical observations, in the last years the need to explore new treatment approaches targeting emotion identification, expression and regulation has emerged, with the aim of fostering the process of recovery, enhancing quality of life (27) and improving long-term outcomes in the ED population (32). Another issue which should not be overlooked is that, notwithstanding patients’ primary psychiatric diagnosis, increasing evidence suggests that alexithymia may play a role as risk factor for suicide, often mediated by depressive symptoms (37, 38). Biological correlates of the relationship between alexithymia and suicidal behaviors have been suggested, including for instance homocysteine dysregulation (39). This evidence further supports the importance to screen for alexithymia in the everyday clinical practice with psychiatric patients, including those suffering from EDs (40). The aim of the current scoping review was to assess treatment outcome in AN in terms of changes in alexithymia as assessed by its most commonly used self-report measure, the Toronto Alexithymia Scale (TAS).

## Methods

A scoping review was conducted in accordance with the Preferred Reporting Items for Systematic Reviews and Meta-Analyses [PRISMA Statement; (41)]. The Medline and Scopus databases were searched on August 30^th^, 2019, and on September 2^nd^, 2019. For Medline, the following keywords were used: [(anorexia nervosa) AND (Toronto Alexithymia Scale)] AND ((therapy) OR treatment). Scopus was searched with the following research string: [ALL (“anorexia nervosa”) AND ALL (Toronto AND Alexithymia AND Scale) AND ALL (treatment OR therapy)] AND [LIMIT-TO (DOCTYPE, “ar”)] AND [LIMIT-TO (LANGUAGE, “English”)].

Two independent reviewers (EG and CG) assessed the articles identified by the above described key words. To be included in the review, studies had to: (a) be clinical trials dealing with patients affected by AN, without limits of age; (b) involve the application of any kind of targeted therapy or treatment; (c) assess alexithymia and the effect of a treatment intervention on alexithymia, using the TAS. Only articles in English were considered eligible. The presence of a control group was not considered an inclusion criterion. Studies which did not match the inclusion criteria described above were excluded. Possible disagreement between reviewers was resolved by joint discussion with a third review author (PZ). Quality of studies was assessed with the Newcastle Ottawa Scale (NOS) (42) by the reviewers who assessed the articles (EG and CG).

Data extracted from the selected studies were recorded in a datasheet using a standardized coding form, including the following categorical and numerical variables: general information about the study (author/s, year of publication, title, journal title, volume, pages, country, study type), sample features (sex, age, BMI, years of illness, diagnosis, treatment; medical and psychiatric comorbidities; sample size, number in experimental group, number in control group, lost at follow-up), setting (inpatient, outpatient…), type of intervention (group, individual, setting…), questionnaires used, study outcome (primary or secondary), main study results.

## Results

### Selection Process

**Figure 1:**
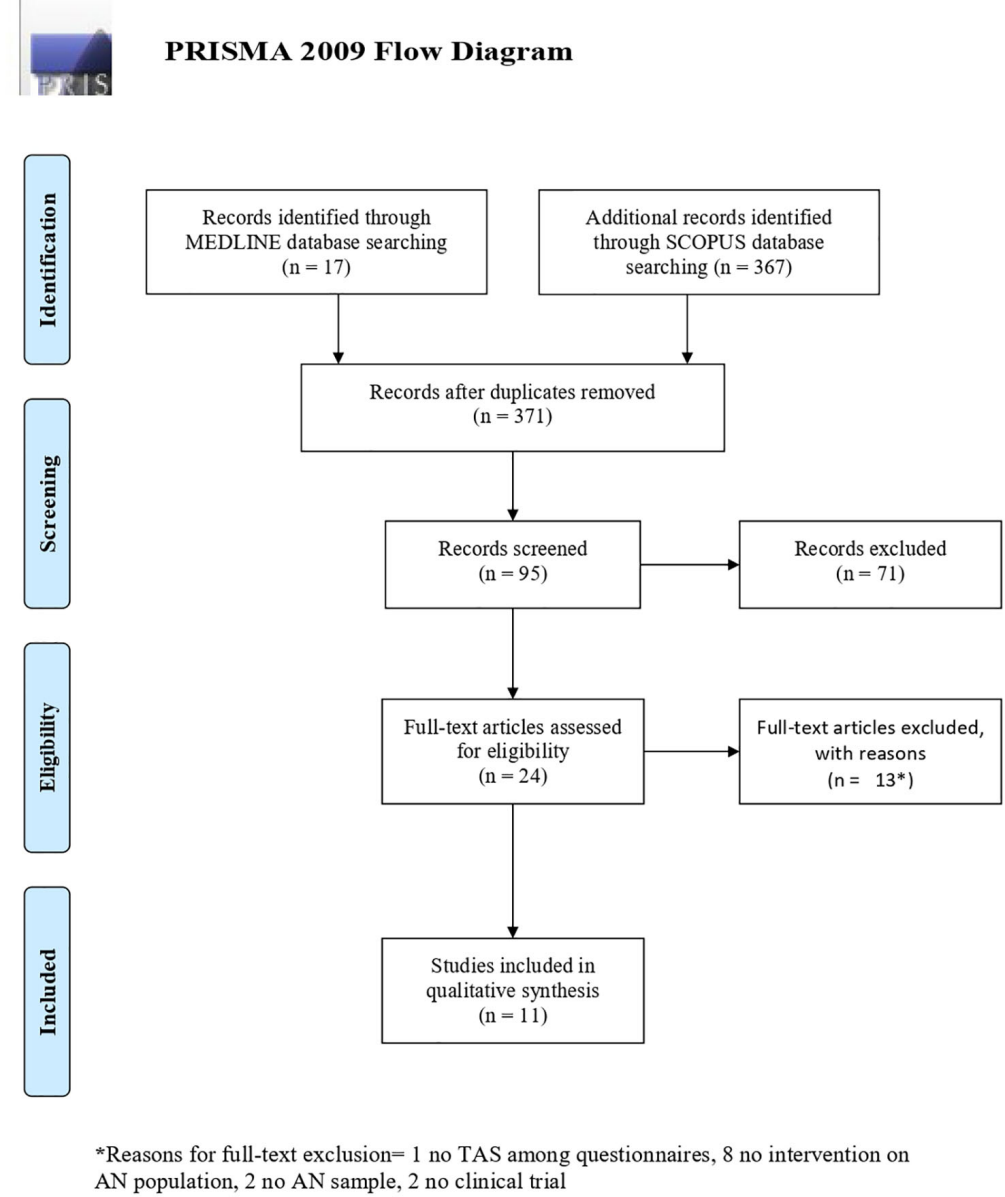
PRISMA 2009 Flow Diagram.

As described in the PRISMA flow diagram (**Figure 1**) (43), the initial search identified 384 titles (17 from Medline, 367 from Scopus); after removing 13 duplicates, titles were screened first, and those clearly not in line with the purpose of the review were excluded (N = 276). Then abstracts were assessed, leading to the exclusion of 71 records, and last full texts were read, eventually leading to the inclusion of 10 papers (2, 15, 32, 44–50) in the qualitative synthesis.

### Quality Assessment

The NOS scores for the included studies ranged from 1 to 6, with a mean score of 3.9 ± 1.59 (SD).

### Description of Study Features

The main features of the selected studies are shown in **Table 1**.

**Table 1:** Main features of the clinical studies included in the review.

Author, year	Sample features	Setting	Intervention assessed	Assessment	Outcome	Main results	NOS
Speranza et al. (2)	N = 102 with complete assessment (initial sample N = 144; directly interviewed N = 109) Gender 100% females Age, mean 21.5 ( ± 5) Diagnosis AN (N = 63) or BN (N = 39) (DSM-IV) BMI n.s. Illness duration, mean 4.3 ( ± 4.8) Comorbidities, anxiety and depressive disorders, substance-related disorders diagnosed with Mini International Neuropsychiatric Interview (MINI).	Multicenter research project involving academic psychiatric hospitals.	Treatments could include (1) pharmacotherapy, any kind, for at least 3 months; (2) Psychotherapy, any kind, for at least 6 months; (3) Hospitalization, either full or partial; (4) Pharmacotherapy + psychotherapy.	Assessment at baseline and 3-year follow-up with: clinical interview BDI-13 CGI MINI MMPI-2 (Negative Treatment Indicators scale) TAS (cutoff ≥56)	Explore the relationship between alexithyimic features and treatment choices in a naturalistic prospective study.	Global improvement of alexithymia and depression. TAS scores not influenced by antidepressants, psychotherapy, or both. Patients received different treatments (number, type) according to their alexithymic profile. Patients with high levels of alexithymia: more treatments and more antidepressants. Patients who became alexithymic during follow-up: more often re-hospitalized; fewer psychotherapies.	4
Tchanturia et al. (15)	N = 33 who attended all 10 sessions and completed both pre- and post-intervention assessments (63% of the sample who completed baseline measures) Gender n.s. Age, mean 24.5 ( ± 8.2) Diagnosis AN (n.s.) BMI, mean 15.1 ( ± 1.95) Illness duration, mean 8 ( ± 7.2) Comorbidities (n.s.)	Inpatient, South London and Maudsley National Adult EDs Service.	CREST: manualized 10-session intervention addressing emotion processing, in individual format. Includes psycho-education, experiential exercises, homework tasks.	RSAS TAS Motivational ruler exploring importance to change and perceived ability to change	Primary: RSAS and TAS scores. Secondary: perceived “importance to change” and “ability to change”; BMI	Decrease of anhedonia (RSAS) and alexithymia (TAS) after CREST. Increase of perceived “ability to change” and of BMI.	3
Giombini et al. (32)	N = 32 who completed the group cycle Gender 87.5% females Age, mean 14.03 ( ± 1.75) Diagnosis AN (DSM-5 criteria) Weight for height %, mean 75.68 ( ± 6.44) Illness duration n.s. 28.1% comorbidities.	Inpatient child and adolescent EDs unit offering multi-disciplinary treatment (individual, family, and group therapy).	CREST-YP: manualized 5-session intervention, weekly, in group format. Includes psychoeducation, experiential exercise, homework.	Semi-structured qualitative interview created *ad hoc*. SQ ERQ-CA RSAS TAS Motivational ruler assessing self-reported importance to change and ability to change. Data collected at the beginning and at the end of the cycle.	Suitability of CREST-YP. Experience of YP receiving CREST-YP. Emotional functioning pre- and post- intervention.	Themes reported by IP: Exploring emotions; Emotions and ED; Homework, Suggestions for improvement. Quantitative results only partially supporting qualitative ones: no significant change in ERQ-CA, RSAS, TAS, and motivational ruler scores. CREST-YP is a suitable intervention for YP with AN.	4
Beadle et al. (44)	N = 20 AN patients tested both during starvation and after weight restoration (out of N = 26 tested at baseline); compared to 16 age-matched healthy women at comparable timepoints. Gender 100% female Age in the AN sample, mean 24.4 ( ± 5.5). Diagnosis AN (ANP or ANR) BMI: mean 15.7. Weight restoration defined as a BMI of at least 18.5 (in the 20 patients sample, mean BMI at time 2, 20.16 ( ± 1.24). Illness duration = n.s. Comorbidities n.s.	Inpatient or outpatient day program following hospitalization.	Inpatient eating disorder program: daily cognitive behavioral therapy, group and individual therapy, supervised eating, occupational and recreational therapy, physician supervision.	EAT EDI-3 YBC-EDS HDRS HARS WAIS-IV CPT II Stroop Color and Word Test WCST TAS-20 IRI MMPI-2 (37 items).	Differences (demographic, cognitive, clinical, and personality characteristics) between participants with AN and healthy controls. Differences between the starvation and weight restoration phases within the AN group. Examination of relationships between alexithymia, emotional empathy, and self-regulation, controlling for depression and/or BMI when relevant.	In the group of AN patients, decrease of DIF and EOT from the starvation to the weight restoration phase. No change in DDF. Overall improvement in alexithymia.	6
Ohmann et al. (45)	N = 29 Gender 100% female Age, mean 14.3 (range 13–17) Diagnosis AN (ICD-10 Criteria): N = 22 ANR; N = 7 ANB BMI, mean 15 (range 13–17) Illness duration, mean 7.2 months (range 2–18). Comorbidity in N = 23 cases N = 5 patients (17%) treated with antidepressants. N = 3 had previous psychotherapy.	Eating Disorder Outpatient Clinic, Department of Child and Adolescent Psychiatry, Medical University of Vienna (recruitment between May and September 2003–2006).	A maximum of 40 weekly sessions (90 minutes each) of Multimodal G-CBT program, including 9 modules: therapeutic motivation, psycho-education, individual problem analysis, (teaching of) problem solving strategies, soft and communication skills, hedonistic training, elements of awareness, body and schema psychotherapy. Family sessions once monthly.	YSR (baseline and 9-months after) At baseline and 3-6-9 and 12-months follow-up: EDE BDI FBA ASW MUM EV-H EV-A MUM-SOC SPS SIAS BIKS TAS	To assess changes of the psychopathological measures during the course of G-CBT and 1-year follow-up. To investigate whether G-CBT is effective for the treatment of adolescent AN and whether emotional risk factors might complicate the course of the disorder. Hypothesis: patients with less disturbed handling of emotions would have a better treatment outcome.	Severe and multiple emotional deficits, difficulties of emotional control, problems in self-confidence and self-efficacy in AN. Especially in patients with poor outcome: problems in intrafamilial communication and expression of emotions. Alexithymia and SOC were disturbed and resistant to change in all patients. Problems in handling, detecting, and expressing emotions are involved in promoting and maintaining anorexic behavior, and are resistant to change, especially in the patients with poor outcome.	4
Iancu et al. (46)	N = 30 soldiers with ED Gender 90% female. Age, mean 19.5 (range 18–21). Diagnosis N = 10 AN (33%); N = 15 BN (50%); N = 5 EDNOS (17%) (DSM-IV). BMI n.s. Illness duration n.s. Comorbidities, No Axis I comorbidity 90% had a personality disorder (mostly borderline or narcissistic) according to the SCID-II questionnaire	Eating Disorder Clinic at the Zeriffin Mental Health Clinic, Israel Defense Forces (2001–2003).	Weekly group meetings (90 minutes) 6 months (lead by a social worker with a social worker and a dietitian as cotherapists) Meetings included: psychoeducation; cognitive behavioral therapy combined with a dynamic approach.	Assessment before and after the intervention with: EDI-2 EAT-26 TAS-26 DES	To examine the efficacy of treatment program. To evaluate the rate of alexithymia and dissociation proneness in the sample (before and after treatment).	Treatment was associated with a significant improvement in eating symptoms (50% decrease in the EAT-26 and EDI- 2 scores), but not with a significant decrease on the DES and TAS-26 scores.	5
Lundbad et al. (47)	N = 30 Gender 100% female Age range 25–40. Diagnosis AN and/or BN (type of eating disorder described by patients and EDI-2 questionnaire) BMI n.s. Illness duration, > 5 years Comorbidities, n.s.	Referred from the Department of General Psychiatry, Sahlgrenska University Hospital (Anorexia and Bulimia Clinic for Adults), Sweden.	Psycho-pedagogic method facilitating the ability of patients to cope with negative feelings: 8 weekly written and oral sessions teaching and coaching about emotional/affective status, in a group format.	TAS	TAS scores changes after intervention.	Significant reduction (pre- to post- intervention) of alexithymia (TAS). Alexithymia negatively correlated with education; no correlation with illness duration, weight loss, depression, general psychoneurotic pathology.	1
Becker-Stoll and Gerlinghoff, (48)	N = 47 (N = 18 AN; N = 25 BN; N = 4 EDNOS) Gender 100% female Age, mean 21.7 ( ± 3.4; range 16–30). Diagnosis AN and BN (DSM-IV criteria). BMI, mean in AN 16.2 ( ± 1.5); mean in BN 22.8 ( ± 6.1) Illness duration n.s. Comorbidities n.s.	Day Hospital at the TCE, Max Planck Institute of Psychiatry, Munich (2001).	TCE: three-phase treatment program consisting of a 4-week outpatient motivation phase, a 4-month day hospital phase, and a 4-month outpatient follow-up treatment phase. Group psychotherapy includes cognitive-behavioral, psycho-educational and interpersonal interventions. Exercises to support body acceptance, including video-confrontation, relaxation techniques, and dance therapy.	Pre- and post-treatment assessment with: EDI (total score) TAS-20	To determine whether a 4-month treatment program has an influence on alexithymia in ED patients. To assess whether alexithymia predicts treatment outcome in the ED population.	High levels of alexithymia in AN and BN patients, without any difference in TAS scores between AN and BN. TAS scores decreased from pre- to post- treatment as well as EDI ones. Baseline scores for alexithymia did not predict post-treatment outcome.	4
Elzakkers et al. (49)	N = 70 (N = 56 assessed at 1-year follow-up; N = 50 at 2-year follow-up) Gender 100% female Age, mean 27.3 ( ± 9.7) Diagnosis, % ANR/ANP 49/51 (Severity of eating disorder symptoms was rated with the EDEQ BMI, mean 15.5 ( ± 1.9) Illness duration, mean 8.6 ( ± 8.1) Comorbidities n.s.	National specialist center for the treatment of eating disorders.	Treatment including individual and group therapies, psychomotor therapy, rehabilitation on and outpatient, day-hospital or inpatient basis.	Baseline, 1- and 2-year follow-up. BMI BDI-II EDEQ IGT MacCAT-T STAI TAS Full remission defined as having: BMI range 18.5–25; resumed menses; no disabling anorectic conditions. Partial remission defined as satisfying 2 out of the 3 criteria described above.	Mental capacity. Relation of the disorder course with psychological variables and decision making.	Full mental capacity group: mild AN at follow-up; improvement in alexithymia score (below the cutoff of 52 for possible alexithymia); improvement of BMI. Diminished mental capacity group: moderately ill category (DSM-5) at follow-up; no improvement of alexithymia; improvement of BMI; higher likelihood of inpatient treatment.	6
Adamson et al. (50)	Individual CREST, N = 66 Gender 100% female Age, mean 25.8 (range 18–53) Diagnosis AN, (DSM-5) BMI, mean 14.8 ( ± 1.3) Illness duration, mean 8 ( ± 8.5) Score above the AQ10 cutoff 32% Group CREST, N = 62 Gender 100% female Age, mean 25.5 (range 18–63) Diagnosis AN, DSM-5 BMI, mean 14.8 ± 1.4) Illness duration, mean 7.6 ( ± 8.3) Score above the AQ10 cutoff 34% Comorbidities, n.s.	Inpatient.	CREST in individual (N = 66) and group formats (N = 62). Individual format: manualized 8 weekly sessions, 40–45 minutes. Psychoeducation and experiential exercises. Group format: 5 weekly sessions, 60 minutes. Optional, participants can drop out at any time.	Assessment before and after individual and group interventions with: RSAS TAS Motivational ruler (ability to change, importance to change) AQ-10 BMI	Effectiveness of CREST interventions. TAS and RSAS scores. ASD symptoms.	Individual CREST: improvement in patients’ alexithymia; increase in motivation; no impact on social anhedonia; significant effect of ASD symptoms on RSAS and TAS scores. Group CREST: increase in motivation; no impact on social anhedonia and alexithymia; significant effect of ASD symptoms on TAS scores.	2

AN, Anorexia Nervosa; ANP, Anorexia Nervosa Purging Type; ANR, Anorexia Nervosa Restrictive Type; AQ, Autism Questionnaire; ASD, Autism Spectrum Disorder; ASW, Assessment of Self-Efficacy; BDI, Depression Inventory; BIKS, Beck Inventory of Cognitive Schemata; BMI, Body Mass Index; BN, Bulimia Nervosa; G-CBT, Cognitive Behavioral Group Therapy; CGI, Clinical Global Impression; CPT II, Conners' Continuous Performance Test-II; CREST, Cognitive Remediation and Emotion Skills Training; CREST-YP, Cognitive Remediation and Emotion Skills Training—Young People; DES, Dissociation Experience Scale; DDF, Difficult Describing Feelings; DIF, Difficult Identifyng Feelings; DSM, Diagnostic and Statistical Manual for Mental Disorders; EAT, Eating Attitudes Test; EDEQ, Eating Disorder Examination Questionnaire; EDI-3, Eating Disorder Inventory; EOT, Externally Oriented Thinking; ERQ-CA, Emotional Regulation Questionnaire for Children and Adolescents; FBA, General Family Questionnaire; HARS, Hamilton Anxiety Rating Scale; HDRS, Hamilton Depression Rating Scale; IGT, Iowa Gambling Task; IRI, Interpersonal Reactivity Index; MacCAT-T, MacArthur Competence Assessment Tool-Treatment; MINI, Mini International Neuropsychiatric Interview; MMPI, Minnesota Multiphasic Personality Inventory; MUM, EV-H, EV-A, Marburg Scale of Euthymic Disorder; MUM-SOC, Marburg Sense of Coherence Scale; n.s., not specified; RSAS, Revised Social Anhedonia Scale; SIAS, Social Interaction Anxiety Scale; SPS, social phobia scale; SQ, Satisfaction Questionnaire; STAI, Spielberger State Trait Anxiety Inventory; TAS, Toronto Alexithymia Scale; TCE, Treatment Center for Eating Disorders; WCST, Wisconsin Card Sorting Test Version 4; YSR, Youth Self Report; YBC-EDS, Yale-Brown-Cornell Eating Disorder Scale.

Details about treatment were reported for the approaches described below; in the other cases, no specific details were described apart from those reported in **Table 1**.

CREST (15, 51–53) is a low-intensity individual manualized treatment for inpatients with severe AN, based on the cognitive interpersonal model. It targets rigid and detail-focused thinking styles but also has a strong emphasis on emotion recognition skills and emotion management and expression. The aim is to help patients learn about the function of emotions (including the communication of needs to self and others), and to improve skills in emotion labeling, identification, and tolerance in self and others.

Lunbad and coworkers (47) described in detail the group psycho-pedagogic method applied at the Anorexia & Bulimia Clinic for Adults at the Sahlgrenska University Hospital. According to the theoretical premise that alexithymia implies an inability to handle negative feelings, eventually requiring a “protective” or “blocking” ingredient (ED symptoms). The aim of the method is to help patients cope with negative feelings.

Group Cognitive-Behavior Therapy (G-CBT) as described by Ohmann et al. (45) includes the following modules: therapeutic motivation, psycho-education, individual problem analysis, (teaching of) problem solving strategies, soft and communication skills, hedonistic training, elements of awareness, body and schema psychotherapy (54). While the primary therapeutic goals are weight gain and improvement of eating behavior, the secondary goal is improved emotional functioning, including alexithymia measures.

Becker-Stoll and Gerlinghoff (48) described a three-phase treatment program (4-week outpatient motivation phase, 4-month day hospital phase, 4-month outpatient follow-up), including group cognitive-behavioral psychotherapy, psycho-educational and interpersonal interventions.

Iancu et al. (46) adopted a group therapy combining a cognitive-behavioral and a dynamic approach; with more detail, the latter proposes a dynamic understanding of ED symptoms as an externalization of an unsolved conflict.

## Discussion

Overall, according to the studies included in the review, alexithymia levels sometimes remain high even after specific treatment, while changes and improvements may occur in other outcome indicators (e.g. body mass index, eating symptomatology, motivation).

Some studies clearly failed to find a positive impact of treatment on alexithymia as assessed with the TAS (32, 45, 46). On the other hand, others supported an improvement of alexithymia after treatment (2, 15, 44, 47, 48), which was represented by CREST (15), a mixed approach including different types of treatment (2, 44), a 4-month day hospital treatment (48), and a psycho-pedagogic intervention (47), respectively (see **Table 1** for more details). Last, mixed findings have been reported by a couple of studies. An improvement of TAS scores was reported in a subgroup of patients with AN (full mental capacity) but not in another (diminished mental capacity) (49). Furthermore, CREST was described as effective in reducing alexithymia when offered in an individual format, but not in a group one (50).

### General Information

All studies but one (2) involved a single center. Most studies were performed in a hospital setting, on inpatients; only four studies included outpatients (44, 45, 48, 49). Samples size ranged from 29 to 168 patients. One study (44) included also a control group, composed by 16 age-matched healthy women.

### Participants’ Features

The studies included in this review involved patients with an age range from 12 to 63 years old; two studies (32, 45) were specifically focused on an adolescent population. Most studies included only female patients, while two (32, 46) assessed both genders. From a clinical standpoint, four of the 10 studies selected for this review involved patients with different types of EDs: AN-R; AN-P; BN; EDNOS (2, 46–48), while six studies included only AN patients (15, 32, 44, 45, 49, 50). More specifically, diagnosis was made according to DSM-IV criteria in three studies (2, 46, 48), to ICD-10 criteria in one study (45) and to DSM-5 criteria in another one (50). In 2 studies (47, 49) the type of ED was assessed using different questionnaires (respectively EDI-2 and EDEQ). The remaining five studies did not specify the diagnostic criteria used (15, 32, 44, 47, 49). Patients’ BMI ranged from 13 to 21.4; illness duration, when specified, ranged from 1 month to more than 5 years. Comorbidities were specified in four studies (2, 32, 45, 46).

### Intervention Features

All studies but two (2, 47) applied cognitive interventions (15, 32, 44–46, 48, 50). More specifically, patients enrolled in three studies (15, 32, 50) were treated with CREST, in a group format; furthermore, Adamson et al. (50) used CREST in an individual therapy setting as well. Seven studies (15, 32, 45–48, 50) included psycho-pedagogic methods to facilitate the ability of patients to cope with negative feelings, particularly associated to body appearance and food assumption. Speranza et al. (2) provided different treatment approaches, such as pharmacotherapy (for at least 3 months), psychotherapy (for at least 6 months), hospitalization (either full or partial), and a combination of pharmacotherapy with psychotherapy. Ohman and coworkers (45) also offered once-monthly family sessions, in the context of 40 weekly sessions of multimodal cognitive behavioral group therapy (G-CBT) program.

### Assessment

All studies but one (2, 15, 32, 45–50) assessed patients with several questionnaires (either self-report measurers or clinician-rated interviews) both at baseline and after the intervention. Beadle et al. (44) tested patients with the TAS only.

### Outcomes

In line with the inclusion criteria adopted, alexithymia as outcome after the intervention was assessed by all studies (2, 15, 32, 44–50). Moreover, four studies (2, 45, 46, 48) evaluated its possible role as predictor of treatment outcome in the AN population.

### Strengths and Limitations

The current paper provides an up-to-date review of the literature on alexithymia and treatment outcome in AN. Despite the clinical relevance of alexithymia, the number of published studies about its changes after treatment are somewhat limited; the possibility to compare the results of these studies is hindered by their heterogeneity and by methodological issues. Besides the previous review study by Pinna et al. (55), that focused on the implications of alexithymia as a predictor of treatment outcome in subjects affected by ED, there are no similar studies on interventions for alexithymia in anorexic patients, making the current review useful to the community of researchers in the field of EDs. Some limitations of this scoping review should be underscored: first, only two databases (i.e., Medline and Scopus) were searched to identify relevant articles written in English language, with potential loss of valuable additional information. The restriction to published studies could *per se* represent a bias in systematic reviews of effectiveness. Despite we focused on TAS as outcome measure in patients with AN undergoing any kind of treatment, samples sometimes included also patients with other EDs, and it was not always possible to rule out results pertaining only those with a diagnosis of AN. Last, while we focused on the TAS as the most used tool for the assessment of alexithymia, and this ensures the homogeneity of results, this choice obviously lead to the exclusion of all those studies using other rating scales for alexithymia.

On the other hand, we adopted restrictive inclusion criteria and applied the PRISMA methodology for the selection process; furthermore, we assessed the quality of the studies included in this review.

Nonetheless, since our aim was to conduct a scoping review of the literature we did not perform a quantitative data synthesis nor meta-analysis. This represents a further limitation of the current study; a meta-analytic approach would certainly represent an interesting research direction for the future to allow drawing clear, evidence-based conclusions about the clinical implications of the findings that have emerged from our research.

## Conclusions

A high rate of individuals with AN achieve incomplete recovery following treatment. The identification of outcome predictors such as alexithymia is crucial, as well as that of treatments specifically targeting these predictors (56–62).

Further studies aimed at a deeper understanding of the actual impact of alexithymia on the outcome of anorexia are warranted, as well as researches exploring alternative treatment strategies for alexithymia in EDs.

## Data Availability Statement

The datasets generated for this study are available on request to the corresponding author.

## Author Contributions

CG and PZ contributed to the conception and design of the work. EG and CG independently triaged the titles and abstracts to remove those that were clearly inappropriate. The remaining papers, to be included, had to satisfy all the predetermined eligibility criteria. Possible disagreements regarding study inclusion were resolved by discussion with PZ. After selection of the relevant studies, EG and CG independently extracted and tabulated data on study design and outcome data using a standard form. CG and EG drafted the manuscript. PZ revised it critically for important intellectual content.

## Conflict of Interest

The authors declare that the research was conducted in the absence of any commercial or financial relationships that could be construed as a potential conflict of interest.
